# Evaluating the citywide Edinburgh 20mph speed limit intervention effects on traffic speed and volume: A pre-post observational evaluation

**DOI:** 10.1371/journal.pone.0261383

**Published:** 2021-12-31

**Authors:** Glenna F. Nightingale, Andrew James Williams, Ruth F. Hunter, James Woodcock, Kieran Turner, Claire L. Cleland, Graham Baker, Michael Kelly, Andy Cope, Frank Kee, Karen Milton, Charlie Foster, Ruth Jepson, Paul Kelly

**Affiliations:** 1 The Scottish Collaboration for Public Health Research (SCPHRP), School of Health in Social Science, University of Edinburgh, Edinburgh, United Kingdom; 2 Population and Behavioural Science, School of Medicine, University of St Andrews, St Andrews, United Kingdom; 3 Centre for Public Health, Queen’s University Belfast, Belfast, United Kingdom; 4 Institute of Public Health, University of Cambridge, Cambridge, United Kingdom; 5 Physical Activity for Health Research Centre (PAHRC), Institute for Sport, Physical Education and Health Sciences, University of Edinburgh, Edinburgh, United Kingdom; 6 Sustrans, Cathedral Square, College Green, Bristol, United Kingdom; 7 Norwich Medical School, University of East Anglia, Norwich, United Kingdom; 8 University of Bristol, Bristol, United Kingdom; Universitat de Valencia, SPAIN

## Abstract

**Objectives:**

Traffic speed is important to public health as it is a major contributory factor to collision risk and casualty severity. 20mph (32km/h) speed limit interventions are an increasingly common approach to address this transport and health challenge, but a more developed evidence base is needed to understand their effects. This study describes the changes in traffic speed and traffic volume in the City of Edinburgh, pre- and 12 months post-implementation of phased city-wide 20mph speed limits from 2016–2018.

**Methods:**

The City of Edinburgh Council collected speed and volume data across one full week (24 hours a day) pre- and post-20mph speed limits for 66 streets. The pre- and post-speed limit intervention data were compared using measures of central tendency, dispersion, and basic t-tests. The changes were assessed at different aggregations and evaluated for statistical significance (alpha = 0.05). A mixed effects model was used to model speed reduction, in the presence of key variables such as baseline traffic speed and time of day.

**Results:**

City-wide, a statistically significant reduction in mean speed of 1.34mph (95% CI 0.95 to 1.72) was observed at 12 months post-implementation, representing a 5.7% reduction. Reductions in speed were observed throughout the day and across the week, and larger reductions in speed were observed on roads with higher initial speeds. Mean 7-day volume of traffic was found to be lower by 86 vehicles (95% CI: -112 to 286) representing a reduction of 2.4% across the city of Edinburgh (p = 0.39) but with the direction of effect uncertain.

**Conclusions:**

The implementation of the city-wide 20mph speed limit intervention was associated with meaningful reductions in traffic speeds but not volume. The reduction observed in road traffic speed may act as a mechanism to lessen the frequency and severity of collisions and casualties, increase road safety, and improve liveability.

## 1. Introduction

Globally, urban transport is an important determinant of population health, with 1.35 million road traffic deaths being reported in 2016, and road traffic collisions costing most countries around 3% of their Gross Domestic Product [1]. The Global Burden of Disease study recorded in 2019 that transport injuries were one of the top 15 causes of death and disability among working age adults in countries with a High or High-Middle Sociodemographic Index [2]. In addition to the direct public health impacts, urban transport also indirectly contributes to air and noise pollution, reduced road safety, physical inactivity and sedentary behaviour [3]. Consequently, researchers, governments, transport planners, and other stakeholders (e.g., road safety and active travel charities) are seeking ways to reduce the detrimental impact of urban transport on public health, as well as other agendas such as climate change.

A common traffic management method is to try and reduce speeds [4]. Speed reduction interventions have been implemented in two main ways: ‘speed limits’ (signage without physical infrastructure); and ‘speed zones’ (signage with physical infrastructure (e.g., chicanes, humps)) [5, 6]. In 2014 an umbrella review investigating the health implications of 20mph zones and limits concluded that residential and area-level schemes (traffic calming 20mph zones) can reduce road traffic collisions, injuries, traffic speed and volume, improve perceptions of safety, and be cost-effective [7]. More recently, a meta-narrative evidence synthesis published in 2019 suggested that whilst the evidence for 20mph zones was established, the effects of 20mph limits was less clear [8]. Additionally, the effects on different socio-economic groups and communities are not well understood [7, 8].

Without the need to install physical infrastructure like speed humps or chicanes, 20mph speed limit interventions are cheaper to implement, making them an attractive option to local governments. However, the absence of these features means the intervention is less coercive, being more akin to guiding rather than restricting driver choice. Hence the uncertainty around whether 20mph speed limit interventions can effectively reduce speeds through changing behavioural norms. Consequently, awareness raising, enforcement and educational activities are important components of 20mph speed limit interventions.

Between 2016 and 2018, City of Edinburgh Council (United Kingdom) increased the proportion of roads in the city with 20mph limits from 50% to 80% [9,10]. The aim of the current study was to describe any changes in traffic speed and traffic volume that followed the implementation of the 20mph speed limit intervention in Edinburgh. Our research objectives were to:

1. Describe the pre-post changes in traffic speed one year after the 20mph speed limit implementation.
2. Describe the pre-post changes in traffic volume one year after the 20mph speed limit implementation.
3. Test whether any changes in speed or volume varied by day of week, time of day, or baseline speeds observed.
4. Investigate the impact of key variables on (any observed) speed reduction over the study period

## 2. Methods

The city-wide 20mph (32km/h) speed limit intervention in Edinburgh, Scotland, was implemented between July 2016-March 2018 [9]. This was a city council policy, with a range of anticipated outcomes including reductions in speed, improved public perceptions of safety, reductions in risk and severity of collisions, and improved liveability [10]. The intervention was implemented in four phases across seven ‘implementation zones’ over the 2-year period (see Table 1) [10, 11]. A network of ‘key arterial routes’ remained at higher speeds (30, 40, 50 and 60mph) in order to facilitate necessary travel while minimising risks [11]. The budget for the intervention was £2.22 million, incorporating signage, a Traffic Limit Order, enforcement, and extensive awareness raising and educational campaigns [12].

**Table 1:** Intervention implementation zones and timetable obtained from the City of Edinburgh council (Scottish Government, 2020) [30].

Zones	Area	Phase	Date	SIMD 2016 (median)**	% Residential	Approximate Area (km^2^)	Urban/Rural Status*
1a	City Centre	1	31 July 2016	3490	16.7%	3.38	100% LA
1b	Rural West	1	31 July 2016	5739	71.4%	143.33	46.4% LA, 40.9% AST, 12.7% AR
2	North	2	28 February 2017	4473	33.3%	16.91	100% LA
3	South Central/East	2	28 February 2017	5630	57.1%	29.43	100% LA
4	North West	3	16 August 2017	6103	71.4%	15.56	100% LA
5	West	3	16 August 2017	3562	88.9%	18.64	100% LA
6	South	4	5 March 2018	4828	80.0%	32.65	99.9% LA, 0.1% AR

*LA: Large urban, AST: Accessible small towns, AR: Accessible rural),

** Scottish index of multiple deprivation.

The ‘Is Twenty Plenty for Health?’ study was a National Institute for Health Research (NIHR) funded project to evaluate the 20mph speed limit intervention in Edinburgh (and also a similar scheme in Belfast, Northern Ireland) [13]. The project had four key work-packages (i) evaluate policy processes and political conditions that led to 20mph implementation, (ii) develop a qualitative understanding of implementation and effects, (iii) assess quantitative outcomes of the implementation, and (iv) conduct cost-effectiveness analysis. The current study was designed to contribute to work-package (iii) by quantifying changes in traffic speed and volume.

To address the research questions, this study describes analyses of repeated (pre-post) measures of traffic speed and volume on the same streets with a 12-month interval. As such, we have described it as an observational, longitudinal evaluation of a natural experiment.

### 2.1 Data collection and data provision

Speed and volume data were collected by Tracsis for City of Edinburgh Council using automatic traffic tube monitors, on 66 streets (equivalent to 3% of the new 20mph network) across the seven implementation zones (see Table 1) and included main, residential, city centre and shopping streets. The selection of the 66 streets for monitoring was made by the City of Edinburgh Council, prior to the commencement of this study, following discussions with the Local Transport & Environment Managers, feedback from a public consultation on 20mph speed limits and by random selection.

Baseline measurement for all streets in all zones took place one-week pre-implementation. The corresponding post measurement took place 12 months later. At each time-point, data collection was conducted over 7 days. The data were collected between 2016 and 2019 to cover the pre- and post- implementation timeframes for each of the 20mph implementation zones.

For this paper two separate datasets were derived from the data provided by the City of Edinburgh Council:

1. Average speed aggregated by street (n = 66), by time of day (96 time-points pre- and 96 post-implementation representing each 15-minute interval in 24 hours) over a 7-day period, including averages for each day of the week. This was also used to create a speed bin (count of speed observations per speed range) dataset.
2. Average volume (7-day averages) aggregated by street (n = 66), for both pre and post timeframes, and average volume by time of day per day of the week over a 7-day period.

### 2.2 Analytical approach

Details of each statistical test, linked to the corresponding research objective, are outlined below:

#### 2.2.1 Objectives 1–3 (changes in speed and volume)

The speed and volume of vehicles pre- and post-intervention were initially assessed using measures of central tendency and dispersion, before being compared using basic t-tests. The changes in speed and volume before and after the speed limits were calculated for the city overall and separately for each 20mph implementation zone and street type category (Main vs. Residential). Summaries based on baseline speeds (<24mph or ≥24mph), time of day (per hour) and day of the week were also created for traffic speed to further explore the overall results. Differences between pre and post mean speeds (and 95% confidence intervals) were calculated using paired-samples t-tests, with significance level set at alpha = 0.05.

#### 2.2.2 Objective 4 (modelling changes in speed and volume)

A mixed effects logistic regression (generalised linear mixed effects model) was constructed to assess the impact of key variables (such as time of day, average speed before the speed limits, and implementation zone) on the odds (or chance) of a reduction in average speed post 20mph speed limits whilst accounting for variation in sampling site (street).

The model was constructed to assess the log odds of an average speed reduction greater than or equal to 0.5 mph for key variables. We selected a threshold of 0.5mph so as to incorporate a measure of meaningfulness in the model estimates. That is, log odds of speed reductions greater than 0.5 mph are more informative than that of reduction of speeds overall.

The dependent variable considered here is a dichotomous variable, with 1 denoting speed reduction greater than or equal to 0.5mph, and 0, otherwise. The explanatory (predictor) variables in the model are time of day (categorical variable with 3 levels), pre-20mph speed range (categorical variable with 4 levels), and zone (categorical variable with 7 levels) (Table 2). Random effects at the level of sampling site (street) are incorporated.

**Table 2:** Predictor variables included in mixed effects logistic regression model.

**Variable name**	**Levels** (with reference category in bold and italics where applicable)
Implementation zones	Zone 1a, Zone 1b, 2, 3, 4, 5, ***6***
Phase of day	***Early morning (midnight to 8*:*00)***, working day (8:15 to 17:30), night (17:45 to 23:45)
Pre-20mph speed range	<20mph, ***20-24mph***, 25-30mph, > 30mph
Speed reduction	Derived from the 7-day average speed and timeframe components of dataset.

Inclusion of random effects at the street level in the modelling process allows us to account for heterogeneity in the observations which are due to the specific street sampled. The street sampled could contribute to heterogeneity in the data and hence impact the model estimates. While we are not interested in the impact of each street on the overall change in vehicular speed (as in the use of a fixed effect), we would like to ensure that this source of variation is accounted for.

The model can be summarised as in Equation A1, where p denotes the probability of average speed reduction (0.5 mph or over), and $\text{log}\left(\frac{p}{1-p}\right),$ the log odds of average speed reduction. Note that the parameters of the model are represented by *β* and the variables by *x*.

**Eq. A1**: **Mixed effects logistic regression model for assessing average speed reduction**

$\text{log}\left(\frac{p}{1-p}\right)=\beta_{0}+\beta_{1}x_{1}+,\ldots ,\beta_{n}x_{n}+{\vartheta \;}_{m}z_{m}$ where p denotes the probability of average speed reduction (0.5 mph or over), *β*_0_ + *β*_0_*x*_1_ +, …, *β*_*n*_*x*_*n*_ + *ϑ*_*m*_*z*_*m*_, the linear predictor, *β*_*n*_, the model parameter associated with variable *x*_*n*_ and *ϑ*_*m*_, the random effects variable for street/site *z*_*m*_.

A brief summary of the project’s findings on road traffic collisions and casualties is provided in the Results section to set the context of the observations made in traffic speed and volume.

## 3. Results

### 3.1 Objective 1: Describe the pre-post changes in traffic speed one year after the 20mph speed limit implementation

**Fig 1:**
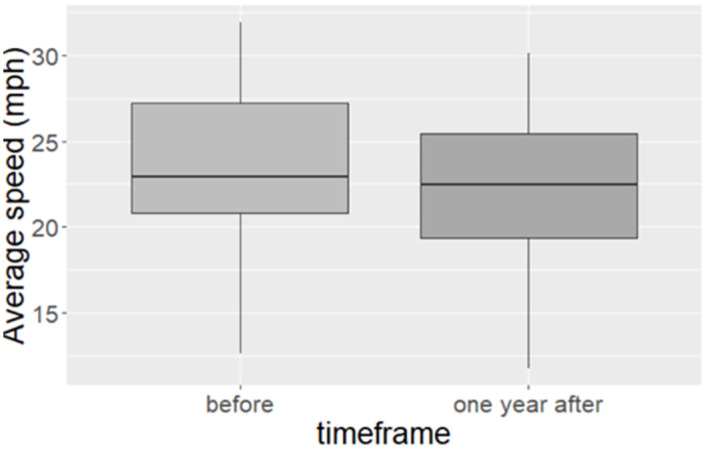
Boxplots for before and after speed distributions for all 66 monitored streets.

The mean and median speeds reduced by 1.34mph (95% CI 0.95 to 1.72) and 0.47mph respectively comparing pre to post data at 12 months. There were also reductions across the distribution of speeds (inter quartile range, and range (maximum to minimum)). The greatest reductions were observed for the maximum speed observed (1.79mph) and the third quartile (1.78mph) (Table 3). A boxplot summary of the speed distributions at a city level before and after implementation is shown in Fig 1. This illustrates that although the change in median speed was small, there was a more marked shift in the distribution of speeds.

**Table 3:** Summary of overall before and after average speed (mph) and volume for all 66 monitored streets.

Statistic (over 66 streets)	Speed in mph (weekly average of speed over every 15-minute time-period per day)			Volume (average vehicular volume/week)		
	Before	After	Difference	Before	After	Difference
Mean	23.63	22.29	-1.34	3,641	3,555	-86
Standard deviation	4.46	3.98	-0.48	2,633	2,592	-41
Median (50th centile)	22.96	22.49	-0.47	3,738	3,391	-347
Q1 (25th centile)	20.84	19.37	-1.47	743	716	-27
Q3 (75th centile)	27.23	25.45	-1.78	5,862	5,860	-2
Minimum	12.59	11.77	-0.82	144	154	10
Maximum	31.90	30.11	-1.79	9,343	9,788	445

**Fig 2:**
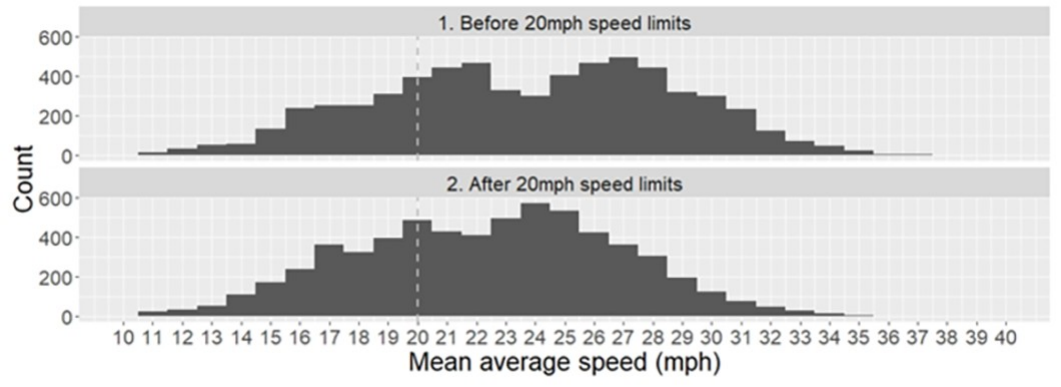
Histogram of average speeds on 20mph streets in the City of Edinburgh for each of the 66 monitored sites (n = 12,672 observations).

These distributions are further presented as frequency plots at 1mph intervals in Fig 2. The speed distribution both before and after the 20mph speed limits appears to be bimodal. Comparison of the distributions shows how the right-hand peak has shifted left in the post implementation distribution indicating reductions in the frequency of higher traffic speeds being observed. It also suggests the left hand peak has shifted from approximately 22mph to 20mph, indicating a reduction at these lower speeds as well.

On further inspection, the observation of a bimodal distribution for traffic speeds is indicative of separate distributions of traffic speed for day and separately, evening-through-to-night time. It is worth noting that reductions in traffic speed were observed across the entire 24-hour period.

Table 4 shows the percentage of observations before and after implementation within specific speed ranges, with relative and absolute changes. After the implementation there were fewer observations in the higher speed ranges “greater than 30mph” and “25-30mph” and more observations in the lower speed ranges “less than 20mph” and “20-24mph”.

**Table 4:** Speed bin summaries showing the percentage of observations (n = 12,672) in four speed bins (ranges) before and after the 20mph speed limit implementation (speed bins derived from the raw speed observations).

	<20mph	20-24mph	25-30mph	>30mph
Before (%)	19.2	35.5	38.1	7.2
After (%)	24.5	44.4	28.0	3.1
Absolute % change	5.3	8.9	-10.1	-4.1
Relative % change	27.6	25.1	-26.5	-56.7

Reductions in mean speed were observed across six of the seven zones (S1 Table). There was a small non-significant increase in Zone 6, though baseline mean speed was already low at 20.25 mph (the second lowest of the seven zones). The largest reduction was observed in Zone 1b, Rural West, 2.41mph (95% CI, 1.36 to 3.46) and the smallest reduction in Zone 4, Northwest, -0.79mph (95% CI, 0.28 to 1.86). The reduction in speed was also slightly greater on main streets (1.59mph, 95% CI, 1.16 to 2.02) than residential streets (1.38mph, 95% CI 0.98–1.78).

### 3.2 Objective 2: Describe the pre-post changes in traffic volume one year after the 20mph speed limit implementation

**Fig 3:**
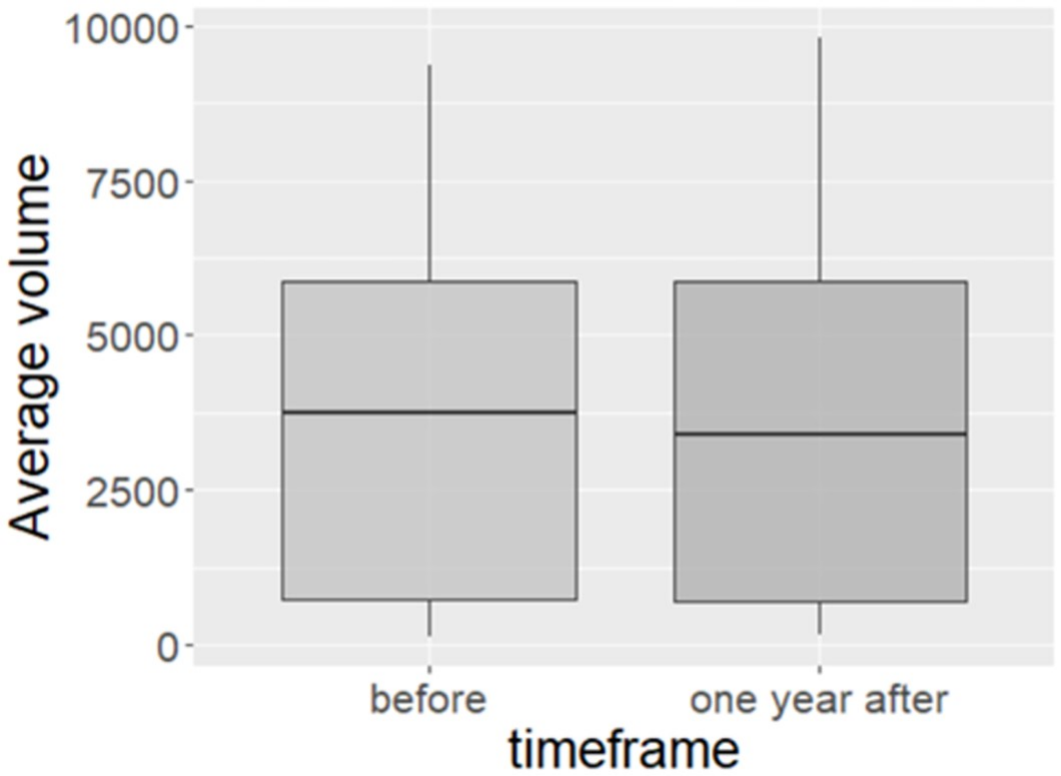
Boxplots for before and after average volume distributions.

In terms of traffic volume, the average number of vehicles passing over the monitor tubes at each site over 7 days prior to the speed limit reduction was 3,641, which reduced by 86 to 3,555 post intervention (Table 3 and Fig 3). Overall, the difference in vehicular volume after the speed limit implementation was minimal (reduction of 2.4%) and non-significant. This was also found for each implementation zone and Main/Residential Street categories.

### 3.3. Objective 3: Test whether any changes in speed or volume varied by day of week, time of day, or baseline speeds observed

As the changes in volume were minimal and non-significant, we only examined variation in speed by day of week, time of day and baseline speed. Reductions in mean speed (mph) were observed on each day of the week (Table 5). These reductions were all found to be statistically significant (p<0.01). The reductions were all similar in magnitude, between -1.16mph (Monday) and -1.48mph (Wednesday).

**Table 5:** Summary of before and after speeds (mean, SD, 95%CI) by day of week (mph).

Category	Before	After	Difference	Standard deviation	Lower CI	Upper CI	p
All data	23.63	22.29	-1.34	1.57	-1.72	-0.95	0.00
Mon	23.48	22.36	-1.16	1.61	-1.55	-0.76	0.00
Tues	23.51	22.05	-1.46	1.68	-1.87	-1.05	0.00
Wed	23.52	22.04	-1.48	1.80	-1.92	-1.04	0.00
Thu	23.53	22.25	-1.23	1.81	-1.67	-0.78	0.00
Fri	23.53	22.16	-1.38	1.70	-1.79	-0.96	0.00
Sat	23.83	22.36	-1.47	1.64	-1.87	-1.06	0.00
Sun	23.91	22.58	-1.30	1.83	-1.75	-0.84	0.00

**Fig 4:**
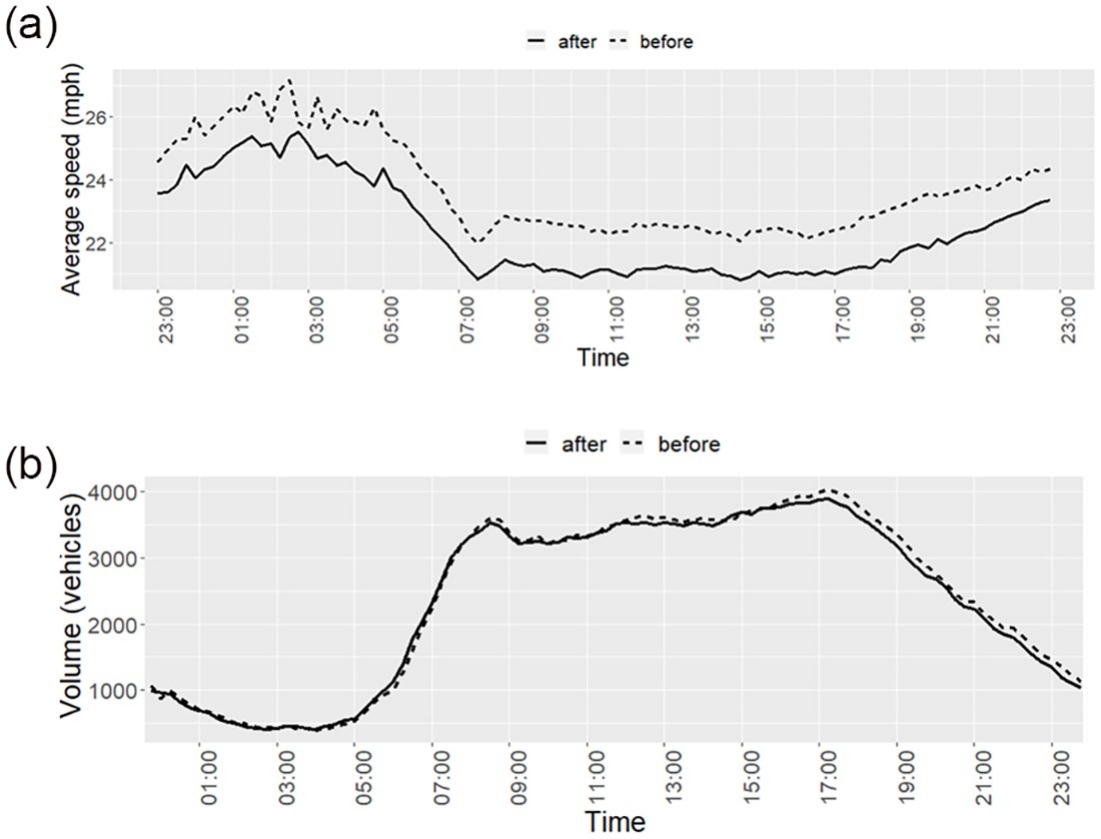
a. Average speeds on 20mph streets in Edinburgh by time of day (from 0:00 to 23:45 by 15-minute intervals). b. Traffic volume on 20mph streets in Edinburgh by time of day (from 0:00 to 23:45 by 15-minute intervals).

When the times were categorised into periods of the day (early morning, working day, and night), the reductions in average speed were again found to be very similar in magnitude. Specifically, the average pre-post difference in speed for early morning, night, and working day was found to be -1.29mph (95%CI:-1.43 to -1.16), -1.36mph (95%CI:-1.45 to -1.26), and -1.35mph (95%CI:-1.42 to -1.28) respectively. Fig 4a shows the change in average speed with time of day treated as a continuous variable. Here it is apparent that relatively higher average speeds are observed between the times 01:00 and 06:00.

Conversely, when considering traffic volume, we note that traffic volume was relatively lower during 01:00 and 06:00 (See Fig 4b). There is an inverse relationship between traffic speed and volume in the data (a correlation of -0.97 before- 20mph speed limits and -0.98 after-20mph).

Reductions in mean speed (mph) were observed for the streets with pre-implementation speeds within the categories <24mph and ≥24mph (Table 6). These reductions were both found to be statistically significant (p<0.01). A larger reduction was observed for the > = 24mph category (reduction of 2.03mph) than for the <24mph category (reduction of 0.72mph).

**Table 6:** Summary of before and after speeds in mph (mean, SD, 95%CI) by pre-implementation speed (mph).

Category	Before	After	Difference	Standard deviation	Lower CI	Upper CI	p
All data (n = 66)	23.63	22.29	-1.34	1.57	-1.72	-0.95	0.00
<24mph (n = 35)	20.09	19.37	-0.72	1.62	-1.28	-0.16	0.01
≥24mph (n = 31)	27.63	25.60	-2.03	1.19	-2.47	-1.60	0.00

#### Objective 4: Which variables are associated with greater odds of meaningful reductions in speed (greater than 0.50 mph) 12 months post-intervention?

Overall, the model results quantify the odds of speed being reduced (post-20mph speed limits) for key variables (Table 7). We note, in particular, that speed reduction greater than 0.50mph is increased by 27% (odds ratio: 1.267) when considering streets with average speed greater than 30mph pre-20mph speed limits (vs streets with pre-20mph average speeds between 20 and 24mph). Note that if the 95% confidence interval (as shown in Table 7) contains 1, the effect under consideration is not statistically significant (at alpha = 5%).

**Table 7:** Results for fixed effects (parameters of interest) of mixed effects logistic regression model for quantifying the odds of speed reduction.

Parameter	Level	Odds Ratio	Lower 95% CI	Upper 95% CI
(Intercept)		1.560	1.261	1.929
Zone	Zone 1a—City Centre	1.250	0.948	1.649
	Zone 1b—Rural West	1.450	1.089	1.931
	Zone 2—North	1.136	0.895	1.443
	Zone 3—South Central/East	1.126	0.880	1.441
	Zone 4—Northwest	0.961	0.728	1.267
	Zone 5—West	1.221	0.938	1.589
	Zone 6—South	ref		
Phase of day	Early morning	ref		
	Working day	1.148	1.123	1.175
	Night	1.087	1.062	1.113
Initial average speed on road	<20mph	0.744	0.718	0.772
	20-24mph	ref		
	25-30mph	1.210	1.172	1.249
	>30mph	1.267	1.200	1.340
**R** ^**2**^		0.178		

It is important to note that our analyses indicated that there was a decrease in road traffic collisions [14, 15] and casualties [15]. In particular, a 40% decrease in collisions was found, along with a 39% decrease in casualties.

## 4. Discussion

Twelve months after a 20mph speed limit intervention across the city of Edinburgh, Scotland, mean speeds were observed to have reduced by 1.34mph (95% CI, 0.95 to 1.72) representing a 5.7% change. Importantly the distribution of overall speeds also shifted. The proportion of vehicles driving at speeds greater than 25 mph at 12 months post-intervention was 14% points lower. Taken together this is consistent with a significant public health impact [16]. The changes in volume of traffic (86 fewer vehicles per week (95% CI: -112 to 286)) were relatively smaller (2.4% change) and there is uncertainty on the direction of effect. This evidence does not indicate an effect on traffic volume of public health significance at 12 months post-intervention.

Reductions in speed were observed throughout the day and across the week. Importantly, larger reductions in speed were observed in the upper tail of the distribution of speeds, especially vehicles travelling over 30mph where the risk of injury or death from a collision is markedly increased [8]. There were differences by implementation zone, with zones that received 20mph earlier generally showing larger reductions in speed; of the three later zones there were non-significant reductions (Zone 4) and increases (Zone 6) in speed. This may be indicative of a change in the driving norms across the city. In the last two implementation zones to become 20mph, the pre-implementation average speed was already close to 20mph (Zone 5, 20.14mph, Zone 6, 20.25mph). The largest reduction (2.41mph) was observed in Zone 1b which was the most rural of the implementation zones. The finding that speeds were higher at night is consistent with speed limit interventions being less coercive than 20mph zones.

The small non-significant change in volume could indicate that the Edinburgh 20mph limit intervention did not have a meaningful impact on modal choice. Anticipated outcomes of 20mph limit interventions include an increase in active travel and liveability, through people finding the roads safer to cycle on and/or walk near, potentially choosing to leave their car at home.

The small change in volume is an indication that the significant change in speed is due to a shift in driver behaviour, rather than the reduction in the number of journeys. This is also supported by the fact that the correlation between traffic speed and volume remained the same before and after the intervention.

Repeat cross-sectional surveys of public attitudes to the Edinburgh 20mph scheme identified higher levels of support for the policy and in rule following after it had been implemented, but no change in perceptions of safety [17]. This might offer an explanation for the reduction in speed, but the lack of marked change in vehicle volume. While people altered their driving behaviour (to drive slower), they did not on the whole switch mode as they did not feel that cycling or walking had become safer. It may be that safety is not the driving force for mode choice in Edinburgh, or perhaps it takes longer than 12 months for safety perceptions and subsequent mode choice to change.

A number of previous studies have sought to quantify or report the impact of 20mph interventions on traffic speed. In our 2019 review, grey literature was identified that reported uncontrolled before and after evaluations of four UK based 20mph speed limits [8]. All four evaluations reported reductions in average speed due to 20mph limits implementation. Reductions varied between Manchester (0.7mph reduction) [18], Bristol (2.7mph reduction) [19], Edinburgh (pilot scheme) (1.9mph reduction) [12], and Portsmouth (1.3mph reduction), with the Edinburgh pilot scheme and Portsmouth reporting greater reductions on roads with higher baseline speeds [20]. In a recent evaluation of twelve case study schemes in England, Atkins and Maher (2018) reported that median speeds had fallen by 0.7mph in residential areas and 0.9mph in city centre areas [21]. They also reported that faster drivers had shown greater reductions in speed.

Cairns et al.’s 2014 umbrella review of 20mph intervention effects reported a 9 mph reduction across 3 areas in UK [22] and that 85% of vehicles were travelling under 30km/h (20mph) post-intervention in The Netherlands [23]. However, as these interventions were traffic calming measures and infrastructure (20mph zones) the larger magnitude of effect is not directly comparable.

The size of the reduction in speed observed in Edinburgh is consistent with previous studies and reviews of 20mph limits, although it is not as large as the reduction observed in some places. Some variation in the size of reduction is to be expected due to differences in context and study design. For example, the greater magnitude of reduction in Bristol (2.7mph) might be explained by higher baseline speeds (27.1mph compared to 23.6mph in Edinburgh), the longer implementation/study period (2–3 years compared to 12 months), and differences in data collection and analysis (quasi-stepped wedge design and reported an adjusted reduction compared to unadjusted pre-post analysis) [19, 24]. In addition, the Edinburgh intervention increased coverage from 50% to 80% of streets [10, 11] and intervention effects may have been larger if initial coverage had been lower. There may also have been differences in implementation, enforcement, and ultimately compliance between studies that have not been recorded or reported. There is little literature for comparison in terms of effects on traffic volume, or factors associated with the odds of reduction in average volume.

There are several strengths and limitations to consider when appraising these findings. In terms of limitations, it was not possible to include data from comparison or control streets in the analysis, as the data utilised were collected by the local council rather than within a research project where control site data collection may have been funded. If this were possible it could have helped to identify the independent effects of the intervention. The analysis here cannot rule out that the changes in speeds observed could have happened anyway (secular trends), or how much of the observed changes can be attributed to the intervention (rather than other events or interventions within the timeframe under analysis).

The assessment of the feasibility of a control or comparison site for research of 20mph limits can be potentially complicated. Should controls be identified within the same city, or would contamination be inevitable? If selected in another city, the impact of extraneous variables present in one site but not the other would need to be accounted for. It is worth reflecting that in a dynamic and complex system such as a city, so many variables change over a 12-month period (e.g., road works, safety campaigns, police enforcement, parking availability, etc.) that even a controlled design and analysis would be subject to many uncontrolled (and often unknown) variables.

The use of council collected data was both a strength and limitation. It meant that research costs were substantially lower, and that baseline data for implementation Zones 1, 2 and 3 were available, as these adopted 20mph limits before the study was funded. However, it meant there was no influence over volume, location, or timing of data collection, which are all potential sources of bias. The council selected 66 streets, but a larger sample could have given better representation of the city, and at a zone level. The automatic counter data are themselves objective (even if placement was a subjective choice) and provide a high number of observations which is a strength. It is also important to recognise that the implementation zones are represented by different numbers of streets, proportions of main to residential street categories, geographical areas, traffic volumes, density of road networks, and population size. For example, Zone 6 was represented by only 5 streets.

### 4.1 Implications for policy and future research

The findings of the current study contribute to the growing body of evidence on the effectiveness of speed limit interventions, which are likely to be more attractive to policy makers than ‘zones’ for their reduced implementation costs. While the reduction in speed was not as large as that observed for 20mph zones, future research will need to explore the cost-effectiveness of 20mph speed limit interventions. Previous research has suggested that a 1mph reduction in speeds equates to approximately a 5% reduction in traffic related injuries [25]. This means that the reductions observed in Edinburgh are likely to be of public health relevance at a town or city level. Importantly, the results suggest a reduction in the higher speeds within the distribution. Elvik (2019) has shown how it is this change in the distribution of speeds, reducing the number of higher speeds, even with ‘slight’ changes in overall average speed, that predict important reductions in mortality risk [26]. An in-depth investigation into the interplay between time of day, traffic volume and speed and road traffic collisions and casualties would advance our understanding of the dynamics involved.

This present study does not provide definitive information to explain “how” 20mph speed limits lead to a reduction in average speeds and an overall trend for reduction across the speed distribution. Previous qualitative work suggested a combination of self-enforcement, copy-cat behaviour, and “pace car” type effects may explain how these limits work [27]. This was supported by our survey work assessing perceptions of the 20 mph limits in Edinburgh [17]. Tapp et al. (2016), described vicious and virtuous circles in this regard [28]. Public awareness was likely raised by 20mph road signage and markings and information campaigns at the time of implementation, and in line with the required legislation written for the scheme to be introduced, it was legally enforceable [9]. Participants in focus group studies as part of the wider “Is Twenty Plenty for Health?” echo these potential routes to change in speed and public health outcomes in Edinburgh and Belfast [29]. However, rigorously studying all the steps in this causal chain to provide definitive evidence is likely to be complicated and expensive [15].

Further research is needed into the impact of 20mph limit interventions on vehicle volume and travel mode choice. These outcomes are likely to be important for achieving broader public health benefits through shifts from sedentary to active travel behaviour as well as reducing climate change. Finally, analysis of effects by indicators of equality, such as the Scottish Index of Multiple Deprivation (SIMD) were not performed. There therefore remains an evidence gap around whether impacts on speed alter inequalities. Such analysis is challenging and will require well-designed methods. For example, measures of driver socioeconomic position cannot be captured by sensors like pneumatic tubes. Matching geolocation of counter data to postcode SIMD would be a very coarse indicator. Is the deprivation status of the street where speed is measured more or less relevant than the street where the journey started and ended? Vehicle telematics and smartphone-based data collection may make these studies more feasible in the future.

## 5. Conclusions

The identification of effective interventions to reduce traffic speeds and related harms is a policy priority. This study used a pre-post evaluation design to assess the impact of the 20mph speed limit intervention in Edinburgh, Scotland. We showed a reduction in speeds across a range of metrics and variables. These reductions are of a magnitude that means they are likely to have public health relevance especially since the observed reduction in traffic speed was accompanied by reductions in road traffic collisions and casualties. Future research is required to understand mechanisms and inform more effective implementation, in addition to assessment of health economic value.

## Supporting information

S1 Table Summary of average speed (mph) overall and by 20mph implementation zone. (DOCX)
