# Supplementary material for: Evaluating the citywide Edinburgh 20mph speed limit intervention effects on traffic speed and volume: A pre-post observational evaluation
Source: PLoS One. 2021 Dec 31;16(12):e0261383. doi: 10.1371/journal.pone.0261383 (PMC8719778; doi:10.1371/journal.pone.0261383)
Supplement: S1 Table — (DOCX) [file pone.0261383.s001.docx]

**Summary of average speed (mph) overall and by 20mph implementation zone**

| **category** | **difference** | **before** | **after** | **% diff.** | **sd** | **C.I.lwr** | **C.I.upr** | **p** |
| --- | --- | --- | --- | --- | --- | --- | --- | --- |
| all zones | -1.34 | 23.63 | 22.29 | -5.67 | 1.57 | -1.72 | -0.95 | 0 |
| zone 1a | -2.07 | 24.14 | 22.07 | -8.57 | 1.58 | -3.54 | -0.61 | 0.01 |
| zone 1b | -2.41 | 23.84 | 21.43 | -10.11 | 1.00 | -3.46 | -1.36 | 0 |
| zone 2 | -1.33 | 23.53 | 22.20 | -5.65 | 1.57 | -2.11 | -0.55 | 0 |
| zone 3 | -1.51 | 25.40 | 23.89 | -5.94 | 1.32 | -2.28 | -0.75 | 0 |
| zone 4 | -0.79 | 26.54 | 25.75 | -2.98 | 1.15 | -1.86 | 0.28 | 0.12 |
| zone 5 | -1.18 | 20.14 | 18.97 | -5.86 | 1.39 | -2.24 | -0.11 | 0.03 |
| zone 6 | 0.41 | 20.25 | 20.66 | 2.02 | 2.40 | -2.57 | 3.39 | 0.72 |
| Main | -1.59 | 24.26 | 22.68 | 1.46 | -1.16 | -2.02 | -1.16 | 47 |
| Residential | -1.38 | 23.61 | 22.23 | 1.60 | -0.98 | -1.78 | -0.98 | 19 |
